# Oxytocin impacts top-down and bottom-up social perception in adolescents with ASD: a MEG study of neural connectivity

**DOI:** 10.1186/s13229-022-00513-6

**Published:** 2022-09-05

**Authors:** Adi Korisky, Ilanit Gordon, Abraham Goldstein

**Affiliations:** 1 The Gonda Multidisciplinary Brain Research Center, Bar-Ilan University, 5290002 Ramat Gan, Israel; 2 Department of Psychology, Bar-Ilan University, 5290002 Ramat Gan, Israel

**Keywords:** Autism, Oxytocin, MEG, Connectivity, Gamma, Face perception

## Abstract

**Background:**

In the last decade, accumulative evidence has shown that oxytocin can modulate social perception in typically developed individuals and individuals diagnosed with autism. While several studies show that oxytocin (OT) modulates neural activation in social-related neural regions, the mechanism that underlies OT effects in ASD is not fully known yet. Despite evidence from animal studies on connections between the oxytocinergic system and excitation/inhibition neural balance, the influence of OT on oscillatory responses among individuals with ASD has been rarely examined. To bridge these gaps in knowledge, we investigated the effects of OT on both social and non-social stimuli while focusing on its specific influence on the neural connectivity between three socially related neural regions—the left and right fusiform and the medial frontal cortex.

**Methods:**

Twenty-five adolescents with ASD participated in a wall-established social task during a randomized, double-blind placebo-controlled MEG and OT administration study. Our main task was a social-related task that required the identification of social and non-social-related pictures. We hypothesized that OT would modulate the oscillatory connectivity between three pre-selected regions of interest to be more adaptive to social processing. Specifically, we focused on alpha and gamma bands which are known to play an important role in face processing and top-down/bottom-up balance.

**Results:**

Compared to placebo, OT reduced the connectivity between the medial frontal cortex and the fusiform in the low gamma more for social stimuli than for non-social ones, a reduction that was correlated with individuals’ performance in the task. Additionally, for both social and non-social stimuli, OT increased the connectivity in the alpha and beta bands.

**Limitations:**

Sample size was determined based on sample sizes previously reported in MEG in clinical populations, especially OT administration studies in combination with neuroimaging in ASD. We were limited in our capability to recruit for such a study, and as such, the sample size was not based on a priori power analysis. Additionally, we limited our analyses to specific neural bands and regions. To validate the current results, future studies may be needed to explore other parameters using whole-brain approaches in larger samples.

**Conclusion:**

These results suggest that OT influenced social perception by modifying the communication between frontal and posterior regions, an attenuation that potentially impacts both social and non-social early perception. We also show that OT influences differ between top-down and bottom-up processes, depending on the social context. Overall, by showing that OT influences both social-related perception and overall attention during early processing stages, we add new information to the existing understanding of the impact of OT on neural processing in ASD. Furthermore, by highlighting the influence of OT on early perception, we provide new directions for treatments for difficulties in early attentional phases in this population.

*Trial registration* Registered on October 27, 2021—Retrospectively registered, https://clinicaltrials.gov/ct2/show/record/NCT05096676 (details on clinical registration can be found in www.clinicalTrial.gov, unique identifier: NCT05096676).

**Supplementary Information:**

The online version contains supplementary material available at 10.1186/s13229-022-00513-6.

## Introduction

### ASD, face processing, and oscillatory neural activity

Autism spectrum disorder (ASD) is a neurodevelopmental disorder characterized by difficulties in the social domain, repetitive behaviors, and restricted thinking [1]. In addition to the diagnostic markers for ASD, several studies have shown that individuals with ASD may also present difficulties in face perception and emotional expression interpretation in a variety of tasks [2, 3]. One explanation for this phenomenon suggests that, compared to typically developed individuals (TD), individuals with ASD tend to focus more on the local details of the face instead of automatically perceiving it as one figure [4, 5].

The ability to identify emotional expression from a face requires a widespread neural network, which includes regions such as the fusiform face area, frontal regions, and occipital cortices [6–8], regions that tend to show atypical activation and connectivity patterns in individuals with ASD. Moreover, it has been suggested that the ability to process the information from facial expressions stems not only from the neural activation in these regions but also from the communication between them [9–12]. One way to conceptualize the relationship between face perception and communication between brain regions relates to the “top-down” and “bottom-up” perception. During face processing, one should balance between bottom-up processing, which focuses on the local features of the face, and top-down holistic interpretation by processing the face as one whole shape [13, 14], a balance that was suggested to be atypical in ASD [4, 5].

Using electro-and-magneto-encephalographic (EEG and MEG, respectively), studies have shown that the unique balance between top-down and bottom-up perception is modulated by the gamma-band frequency power and synchronization, a neural oscillation with a frequency between 30 and 100 Hz [15]. While long-range connectivity in low frequencies (alpha band, 8–13 Hz) is often correlated with a priori knowledge and top-down processing [16], the power and connectivity in gamma represent stimulus-related sensory information, local feature processing [17], and cognitive binding of features [18, 19]—all relevant functions for face processing. Indeed, studies have shown that an increase in gamma power and its synchronization between- and within-regions that relate to social processing, can be detected when TD individuals process upright faces compared to inverted faces or other objects [20–22]. In the autistic population, there is accumulating evidence regarding deficits in alpha, beta, and gamma bands’ power and connectivity patterns during social perception [23–30]. Mainly, it has been shown that during the evaluation of social stimuli, autistic individuals tend to present under-connectivity in the low-frequency bands, and over-connectivity in the gamma band, especially in magnetoencephalography (MEG) studies [31].

However, the existing literature regarding oscillatory connectivity in ASD during social perception suffers from both inconsistencies and gaps. First, despite extensive research on high-band frequencies during social perception in ASD, the results are not consistent across the literature. While some studies suggest that deficits in gamma activation and synchronization can serve as biomarkers for autism [32], other studies have shown that during face processing, interhemispheric connectivity in gamma can be either increased [33–35] or decreased [36, 30]. Moreover, the results are highly dependent on the experimental sample, individuals’ age, chosen task, and the type of the analysis [31, 37]. Second, only a handful of studies examined the phase-lag connectivity of other frequencies, such as alpha and beta-band, which relate more to feedback loops and top-down reasoning. In these frequencies, the results are usually consistent, indicating that compared to TDs, individuals with ASD show hypoconnectivity [38, 23, 39, 40, 36] except some studies showing the opposite direction by reporting an increase in alpha connectivity during social processing [29].

Another scientific approach for investigating the top-down/bottom-up balance in ASD focused on the correlations between low and high frequencies using cross-frequencies methods such as phase–amplitude coupling (PAC) [41]. PAC analysis allows evaluating whether the phase of a low-frequency band (usually alpha) in one region is locked to the amplitude of high-frequency bands in another region [42]. It has been shown that during resting-state measurements, individuals with ASD present increased short-range alpha-to-gamma PAC [43, 44]. During social perception, PAC can shed more light on the balance between top-down and bottom-up perception in ASD by examining the relationships between low and high frequencies. Indeed, studies have shown that individuals with ASD present reduced coupling during face perception in ASD for both long- and short-range connectivity, a reduction that was correlated with clinical symptoms severity [40, 27].

Overall, the literature presents differences in the oscillatory functional connectivity during emotional face processing in ASD, compared to TD. We suggest that these differences may underlie the reported imbalance between top-down and bottom-up facial processing in the autistic population and aim to investigate the influence of Oxytocin (OT) administration on said imbalance.

It has been shown that OT, a naturally occurring hormone that relates to social communication in mammals [45], can influence social perception in autistic individuals. For example, it has been shown that in ASD exogenous OT can modulate face perception, attention toward facial features, and identification abilities of emotional facial expressions [46–51]. Although it has been found that the endogenous levels of OT in children are lower than those of TD individuals or adults [52], the influences of exogenous OT are not consistent and depend on the context in which stimuli were presented, individual clinical and psychological parameters, as well as genetic factors such as OT receptor variants or peripheral OT level. Moreover, it is still not clear how OT influences early perceptual stages and whether these possible effects could modulate face perception in ASD.

One possible explanation for the mechanism underlying OT effects, in the context of early perception, emerges from the possible modulation of neural excitation/inhibition (*E*/*I*) ratio. The *E*/*I* model, developed by Rubenstein and Merzenich [53], proposes that individuals with ASD present higher-than-expected neural activations due to an imbalance between local synaptic signals of excitation and inhibition. Based mainly on animal models of ASD, Lopatina et al. [54] suggest that endogenous and exogenous OT can modify the excitation/inhibition (*E*/*I*) ratio through gamma-power modulation and inhibitory GABAergic pathways. Although this theory focused on local circuits, it has been proposed that disruptions in gamma feedforward course can disrupt long-range information transference across brain areas [41, 55]. Thus, it is possible that by modulating the *E*/*I* ratio, OT can enhance neural connectivity and improve social perception abilities among ASD. While several studies have shown that OT indeed influences social perception and social-related neural activity in ASD, most of these studies did not examine oscillatory activity.

The current study aimed to investigate the effects of OT on top-down/bottom-up modulation in youth with ASD during the early phases of face perception. Specifically, using MEG, we measured gamma, alpha, and beta bands connectivity between three specific a priori-defined social-related regions of interest (ROIs): the left and the right fusiform and the medial prefrontal region. These regions specialize in the perception of face and facial expressions [56–58] and can be modulated by OT in both TD and ASD individuals [49, 59, 60, 61]. However, the effects of OT in these regions during the early phases of social attention are still unknown. We hypothesized that in adolescents diagnosed with ASD, OT would modulate top-down and bottom-up processing during social perception by elevating alpha- and beta-band connectivity and reducing gamma-band connectivity between the mentioned ROIs.

This is the first time such an experimental paradigm has been implemented with MEG in youth with ASD, to the best of our knowledge. Thus, although it is preliminary, we believe that the current study can offer a more comprehensive understanding of the mechanism by which OT can modulate different levels of early perceptions during face processing among autistic individuals.

## Methods

### Participants

The study was approved by the Beer Yaacov-Ness Ziona Mental Health Center Ethics Board (Declaration of Helsinki) and was registered as a clinical trial (ID: NCT05096676). Thirty-two adolescents diagnosed with ASD arrived at Bar-Ilan University for two sessions for a randomized, double-blind placebo-controlled trial. Participants were recruited through social media ads and the Bait Echad ASD clinical centers in Israel of the Association for Children at Risk. All participants met the diagnostic and statistical manual of mental disorders (DSM-5) criteria for ASD. Also, clinical diagnosis was confirmed using the Autism Diagnostic Observation Schedule (ADOS-2) [62], a clinical assessment that was conducted at the Bait Echad clinical center prior to the experimental meeting. Furthermore, we ensured that none of the participants had a comorbid intellectual impairment (cutoff was an IQ > 80) using the Wechsler Abbreviated Scale of Intelligence (WASI, [63]).

From the entire sample, two participants were unable to complete the session due to technical issues in the MEG, and six individuals were excluded from the analysis due to a high percentage of artifacts, such as muscle movements (where at least 30% of the trails did not allow sufficient processing due to artifacts). Thus, 24 participants were included in the data analysis.

All participants were males, aged 12–18 years, native Hebrew speakers, and had a normal or corrected-to-normal vision (see Table 1 for demographic details). Before the experiment, participants and their parents underwent a telephone screening interview regarding chronic medical problems, cardiovascular risk factors, CNS disease, other mental illnesses, and the use of prohibited medications (see Additional file 1 for list of approved medications). Participants with intellectual disabilities, impaired vision or hearing, current substance dependence diagnosis, a history of significant head injury or neurological illness, or metallic implants were excluded from the study. At the beginning of each visit, parents were provided an informed consent form, and the participant provided verbal assent.

**Table 1:** Participant characteristics

Variable	ASD (*n* = 24)
Sex (*M*:*F*)	24:0
Age	*M* = 14.01 (SD = 1.63)
WASI	*M* = 45.8 (SD = 8.6)
ADOS-2	*M* = 10.6 (SD = 2.5)

*WASI* Wechsler Intelligence Scale shows subtest standardized *T*-score (*M* = 50, SD = 10), *ADOS-2* Autism Diagnostic Observation Scale 2nd edition comparison score

All participants were paid 500 NIS for their participation in both sessions.

### Procedure

Participants visited the laboratory for two sessions (approximately 1 week apart). In one session, they received OT, and in the other session placebo (PL). The order of the substance administration was random, and the design was double-blind. All substances were intranasal doses of 40 international units of (IU)/mL and prepared by the “Maayan Haim” pharmacy, Israel. Age-dependent doses were adapted to participants’ ages: 13–18 years old adolescents received a dose of 24 IU (3 puffs to each nostril), and younger participants (aged 12 years) received 16 IU.

Forty-five minutes following intranasal administration [47, 64], participants underwent MEG scanning and digital registration of the head position. In each scan, instructions were provided regarding the need to minimize head or body movements as much as possible. We verified that all participants felt as comfortable and relaxed as possible before and during each scan.

### Paradigm

**Fig. 1:**
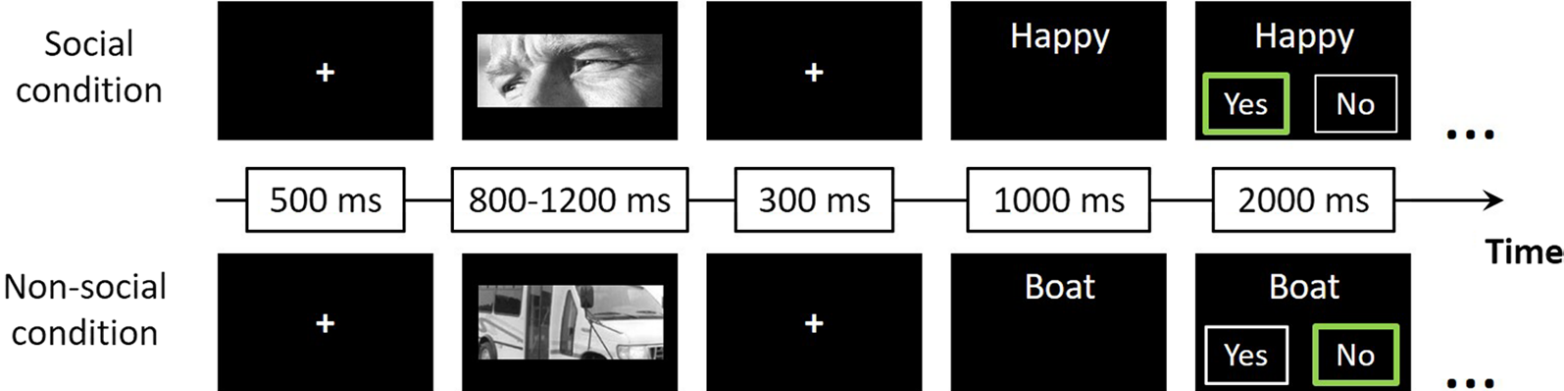
Behavioral paradigm. The task consisted of eight blocks—half contained social stimuli (pictures of eyes during emotional expression, see example above the line) and the other half contained non-social stimuli (pictures of vehicles, see example below the line). Trials began with a fixation cross (500 ms). Next, an image appeared for approximately one second, followed by a single word. Participants were asked to decide whether the word described the image. The three dots at the end of each row mark the continuity of the block

During each session, participants performed a social-related emotion judgment task based on the Reading the Mind in the Eyes Test (RMET-R) [65]. In the task, participants were asked to recognize social (pictures of eyes in a variety of emotional expressions) and non-social (pictures of vehicles) grayscale images. Overall, we presented 160 stimuli–20 different images in each category with four repetitions of each set. The order of the blocks and the order of the pictures inside each block were randomized and counterbalanced across participants. The task lasted approximately 13 min in total (see Fig. 1).

In the MEG, participants were placed in a supine position, and stimuli were presented reflected on a mirror from a 17″ screen located 60 cm above their heads using e-Prime software (version 2.0 professional, Psychological Software Tools, USA).

### Data analysis

#### Behavior analysis

The influence of OT on behavioral performance was assessed using repeated measures analysis of variance (ANOVA), reported with a Bonferroni correction for multiple tests. We conducted a 2 * 2 (OT/PL X social/non-social) design for both accuracy rates and reaction times (RTs, from correct trials only). Finally, we correlated individual parameters (age, WASI, and ADOS) with the effect of OT on each condition.

#### MEG analysis

Due to the different roles that different bands have in face processing and top-down/bottom-up perception, to investigate the influence of OT on connectivity during social perception we conducted connectivity analyses, examining separately long-distance connectivity patterns in alpha (8–13 Hz), beta (14–25 Hz), and gamma (30–100 Hz) bands. Similar to Sun et al. [35], we split the range of gamma frequencies to low- (30–60 Hz) and high-gamma (60–100 Hz) bands. Furthermore, we focused on specific regions of interest (ROI) in all analyses: the left and right fusiform and the medial prefrontal cortex (mPFC, left and right combined). These ROIs were chosen due to their crucial role in face processing and their sensitivity to OT during social-related tasks [49, 56, 59, 50, 57, 58, 61].

#### Preprocessing

Data analysis was conducted using the Fieldtrip toolbox for MATLAB [66] except for the removal of heartbeat artifacts, line power (50 Hz and its harmonics), and excessive external noise, which used in-lab algorithms [67]. Next, data were segmented into 1400 ms epochs: 400 ms before the appearance of the picture and 1000 ms after. We first partly removed bad sensors which presented irregular electrical conductance. Then, by visual inspection, we removed segments containing muscle artifacts after applying a high-pass filter at 60 Hz. Independent component analysis (ICA) was applied (after downsampling data to 300 Hz) to remove eye blinks, eye movements, and remaining heartbeats. Finally, all segments were filtered in the 1–100 Hz range with 10 s of padding. A trial-by-trial visual inspection was conducted to reject unusual trials. High and low bands were inspected separately.

The final data for each individual included only artifact-free trials in which correct answers were received by the participant (OT_social_: *M* = 66.6, SD = 7.37; OT_non-social_: *M* = 63.9, SD = 7.86; PL_social_: *M* = 65.2, SD = 7.42; PL_non-social_: *M* = 64.8, SD = 6.95).

In light of the significant impact that the number of trails has on connectivity measures [68], we equated the number of clean trails for every condition and session by randomly sub-sampling trials according to the participant with the minimal number of trials (in both OT and PL session: *n*_Social_ = 34 trials, *n*_non-Social_ = 34 trials).

#### Connectivity analysis

To evaluate the influence of OT on top-down and bottom-up connections, we assess the connectivity in alpha and gamma bands between the a priori selected ROIs. Long-range connectivity in alpha, beta, and gamma ranges was conducted to determine the connectivity between related social regions. We evaluated the medial frontal regions and the left and right fusiform as one network and examined the overall connectivity in it. First, we conducted a time lock analysis on the preprocessed data for each frequency range (alpha: 8–13 Hz; beta: 14–25 Hz; low gamma: 30–60 Hz; and high gamma: 60–100 Hz). We applied a linearly constrained minimum-variance (LCMV) beamformer to the data, time-locked to the appearance of the stimuli, and computed the overall filter for the source model. Next, to take individual differences in brain topography into consideration, we independently choose the voxel with maximal differences between social and non-social trials for each participant. We then evaluate the neural activity using a virtual channel constructed from the maximum power orientation in each chosen voxel and trial. This was conducted for the entire period in which the stimulus appeared (1000 ms) and was corrected for baseline activity (400 ms before stimulus appearance). Finally, we calculated the spectral connectivity between the ROIs for each frequency range by computing the coherence matrix across the vertices and the averaged coherence score (mean score of upper triangular in that metric). This analysis was conducted twice, once for OT sessions and once for the PL sessions. Furthermore, we used a permutation test (10,000 permutations on the group affiliation—OT/PL) to evaluate the influences of OT in social compared to non-social trials. For each frequency band and condition, we computed the mean coherence score between the fusiform (left and right clusters) and the mPFC. The statistic calculated was the difference between the scores obtained for the OT and PL sessions, for which a null distribution (under the hypothesis that there is no difference in coherence between the sessions) was computed as follows: We resampled the data by conducting a per-subject permutation test on the coherence scores. We swapped the labels of the OT and PL sessions with a probability of 0.5. In each iteration, the coherence score was calculated in the same way as the statistic measurement. For the interaction effect, we normalized, in each session, the network’s coherence score by dividing the score from the social trials from that of the non-social trials. The main effect of OT was examined by averaging the coherence score of social and non-social trials in each session and comparing the average scores. All results in the connectivity analysis (8 *p* values) were false discovery rate (FDR) corrected [69].

In light of the great heterogeneity in the autistic population, we also explored whether OT benefits more with specific individuals by correlating the normalized neural data and individuals’ age and clinical assessment (ADOS and WASI scores).

#### Brain–behavior analysis

To investigate the unique effects of OT on social perception as observed in the interaction that was revealed in the gamma band, we conducted a brain–behavior exploratory analysis. We calculated a new normalized “social perception score” for each individual. Similar to the connectivity analysis, the “social perception score” was composed of the accuracy rates in the social trials divided by the rates in the non-social trials. This score was calculated for the OT session only. We next examined whether the connectivity in gamma bands correlated with the behavioral measurement. For this correlation analysis, we used normalized coherence and behavioral scores.

## Results

### Behavioral analysis

Repeated measures ANOVA was conducted to assess the effects of OT on social perception for both reaction times (RTs) and accuracy rates. Reported RTs analysis included only trials with correct responses (social: *M* = 62, SD = 5.69; non-social: *M* = 66.6, SD = 4.75).

RT analysis revealed a significant difference between conditions (*F*_(1,22)_ = 10.16, *p* < 0.005), as RTs in non-social trials were shorter than those in social trials (*t* = 3.19, *p* < 0.005). No significant effects were found for group (*F*_(1,22)_ = 0.03, *p* = 0.86) or interaction (*F*_(1,22)_ = 0.17, *p* = 0.68). For accuracy rates, no significant effects were found for conditions (*F*_(1,22)_ = 0.36, *p* = 0.55), group (*F*_(1,22)_ = 1.3, *p* = 0.26), or interaction (*F*_(1,22)_ = 1.03, *p* = 0.32). All the reported results are corrected for Bonferroni correction. For descriptive and inferential statistics, see Tables 2 and 3.

**Table 2:** Reaction time differences between the experimental conditions and sessions

Comparison	Mean squares	*F*	*p*	*η*^2^
OT–PL	314	0.03	0.86	0.001
Social–non-social	20,735	10.16	0.004	0.316
Interaction (session × condition)	340	0.174	0.681	0.008

Means are given in milliseconds. All the reported results are corrected for Bonferroni correction

**Table 3:** Accuracy rate differences between the experimental conditions and sessions

Comparison	Mean squares	*F*	*p*	*η*^2^
OT–PL	0.001	0.362	0.553	0.016
Social–non-social	0.006	1.325	0.261	0.054
Interaction (session × condition)	0.004	1.03	0.32	0.043

All the reported results are corrected for Bonferroni correction

However, by correlating the ADOS score with the normalized accuracy rates (social/non-social score) we found a positive correlation between the influence of OT and individual ADOS scores: In individuals with higher ADOS scores, OT increased the differences between social and non-social perception, such that higher accuracy rates were observed in the social trials (compared to PL sessions) (*r* = 0.42, *p* = 0.04). No correlation was found between behavioral performance and WASI scores (*r* = − 0.05, *p* = 0.81).

### Connectivity analysis

Using the permutation test, we explored the difference in the mean coherence scores of the task conditions between the OT and the PL sessions (see Table 4 for descriptive data of the coherence scores in each band and condition and Table 5 for descriptive and inferential statistics).

**Table 4:** Coherence scores from the connectivity analysis for each condition and session

Frequency band	Coherence score OT session	Coherence score PL session
Alpha (8–13 Hz)	*M*_Social_ = 0.653 (SD = 0.03)	*M*_Social_ = 0.632 (SD = 0.028)
	*M*_Non-social_ = 0.654 (SD = 0.04)	*M*_Non-social_ = 0.634 (SD = 0.03)
Beta (14–25 Hz)	*M*_Social_ = 0.66 (SD = 0.04)	*M*_Social_ = 0.63 (SD = 0.034)
	*M*_Non-social_ = 0.66 (SD = 0.038)	*M*_Non-social_ = 0.64 (SD = 0.03)
Low gamma (30–60 Hz)	*M*_Social_ = 0.63 (SD = 0.027)	*M*_Social_ = 0.65 (SD = 0.028)
	*M*_Non-social_ = 0.641 (SD = 0.025)	*M*_Non-social_ = 0.65 (SD = 0.03)
High gamma (60–100 Hz)	*M*_Social_ = 0.633 (SD = 0.026)	*M*_Social_ = 0.645 (SD = 0.029)
	*M*_Non-social_ = 0.637 (SD = 0.02)	*M*_Non-social_ = 0.64 (SD = 0.035)

**Table 5:** Differences in mean coherence scores between OT ad PL sessions in each frequency band

Frequency band	Comparison	*p*	*qFDR*
Alpha (8–13 Hz)	OT–PL	0.012	0.043
	Interaction	> 0.5	–
Beta (14–25 Hz)	OT–PL	0.016	0.043
	Interaction	0.27	–
Low gamma (30–60 Hz)	OT–PL	0.17	–
	Interaction	0.01	0.042
High gamma (60–100 Hz)	OT–PL	0.15	–
	Interaction	0.02	0.08

All the presented statistic was calculated based on the difference between the scores obtained for the OT and PL sessions after 10,000 iterations. Significant *p* values were false discovery rate (FDR) corrected for multiple comparisons

In the alpha band, a main effect was observed for OT, such that higher alpha coherence between ROIs was observed after OT administration (uncorrected *p* = 0.012, qFDR = 0.043, 10,000 permutations*)*. The interaction effect was not significant *(*uncorrected *p* = 0.5, 10,000 permutations).

Similar to the alpha band, in the beta range, a main effect was observed for OT, such that higher beta coherence between ROIs was observed for OT, compared to PL (uncorrected *p* = 0.016, qFDR = 0.043, 10,000 permutations*)*, while the interaction effect was not significant *(*uncorrected *p* = 0.27*,* 10,000 permutations).

**Fig. 2:**
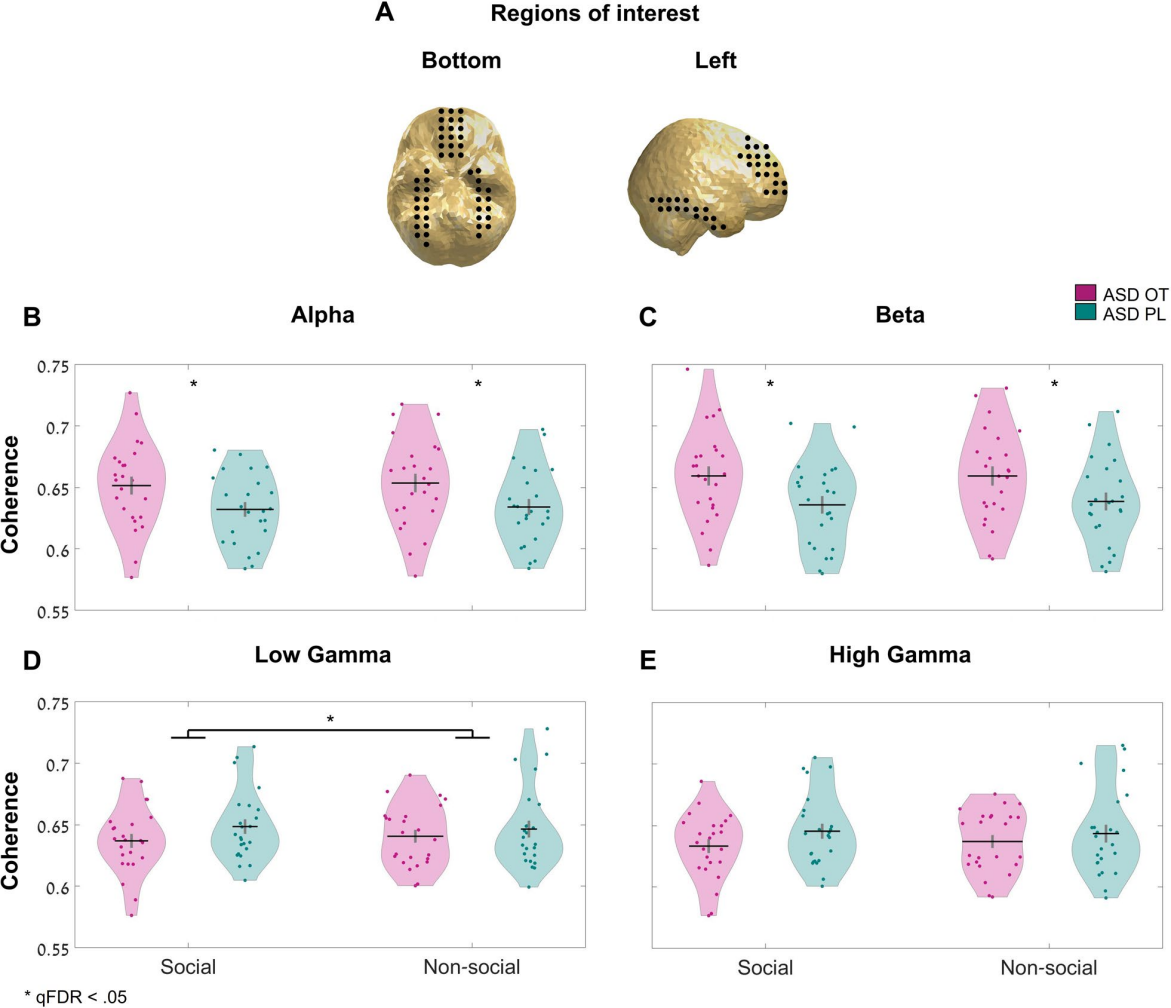
Violin plots of the connectivity between posterior and frontal social-related regions in alpha, beta, and gamma frequencies. **A** Selected regions of interest. Three neural regions were chosen prior to the experiment: left fusiform, right fusiform, and medial frontal cortex. All areas were marked based on AAL atlas’ locations. Black dots represent the selected voxels in each region. For connectivity analysis, we used one voxel from each region. Power analysis was calculated from all the voxels. **B**–**E** The neural coherence score between ROIs, in each frequency band: **B** alpha (8–13 Hz); **C** beta (14–25 Hz); **D** low gamma (30–60 Hz); and **E** high gamma (60–100 Hz). OT and PL sessions are represented as separate lines. Asterisks represent a significant result (*p* < .05) after FDR correction. Dots represent individual data. Black horizontal lines represent the mean. Vertical lines represent mean values $\pm 1$ SE

However, in gamma band the interaction effect was significant in low (uncorrected *p* = 0.01*,* qFDR = 0.0427, 10,000 permutations) and marginally significant for high gamma (uncorrected *p* = 0.02*,* qFDR = 0.08*,* 10,000 permutations). As can be seen in Fig. 2, the coherence in gamma was lower after OT administration, a reduction that was larger in the social, compared to the non-social trials (Social_OT-PL diff_ =  − 0.02; Non-Social_OT-PL diff_ =  − 0.006). No significant main effects for OT were observed for low or high gamma (low: uncorrected *p* = 0.17; high: uncorrected* p* = 0.15, 10,000 permutations).

### Brain–behavior correlation

**Fig. 3:**
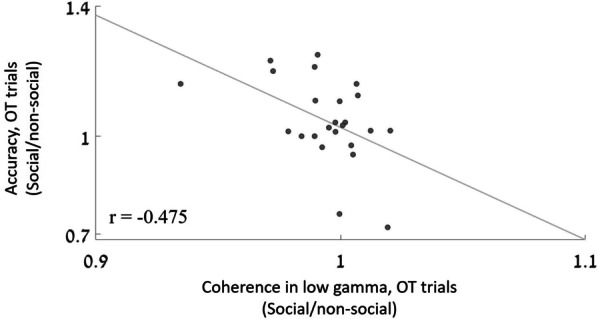
Brain–behavior correlation. A significant negative correlation was observed between low-gamma-band coherence and participants’ performance. The *X*-axis represents normalized coherence—the connectivity between the fusiform and mPFC in the social trials divided by the connectivity in the non-social trials. *Y*-axis represents normalized behavioral performance—accuracy rates in social trials divided by the rates in the non-social trials

To evaluate the correlation between the unique neural influence of OT during social processing and the behavioral performance, we conducted an exploratory correlation analysis. We investigate the correlation between the strength of the coherence in the low-gamma band, where we found a differential effect of OT during social versus non-social perception, and the accuracy rates in the task. Namely, we correlated the coherence score to the ratio of change in the behavioral performance between social and non-social stimuli by dividing the accuracy rates from the social trials by those without the social stimulus. A negative correlation was observed between the strength of the low-gamma connectivity and individual accuracy rates (*r* =  − 0.475, *p* = 0.02): Low-gamma coherence between the fusiform and the mPFC correlated with better performance in social compared to the non-social trials (see Fig. 3).

## Discussion

The current study examined the effects of a single administration of OT on neural oscillations in adolescents diagnosed with ASD in order to assess its impact on top-down and bottom-up processing in this population. Specifically, we examined the influence of OT on the neural coherence of alpha, beta, and gamma bands during face perception in three ROIs: left and right fusiform and mPFC.

Our results show that OT modified social-related posterior-frontal connectivity in two opposite patterns which depend on the examined frequency band. In the lower range of frequency bands, alpha and beta, OT (compared to placebo) increased the connectivity for both social and non-social stimuli. However, in low gamma, OT induced the opposite effect by causing a decrease in the posterior-frontal connectivity. Moreover, compared to the non-selective increase that has been shown in alpha and beta, in gamma the effect of OT was larger during social trials than during non-social ones. We further show that the decrease in the low-gamma range connectivity, following OT administration, correlated negatively with the success of the individuals in social trials compared to non-social trials. Namely, we show that the lower the connectivity, the higher the ability of the participant to identify social stimuli compared to non-social ones.

One possible explanation regarding the results stems from top-down and bottom-up perspectives. Top-down modulation is characterized by feedback loops from frontal and parietal regions and is thought to be manifested in alpha and beta oscillations [70, 71]. Gamma, on the other hand, is known to reflect bottom-up perceptual processes by passing information in a feedforward manner up through the visual stream [72, 17, 73]. Our results show that a single dose of OT can modulate the balance between bottom-up and top-down processing in ASD for both social and non-social visual stimuli. Several studies have shown that during face processing, autistic individuals tend to process facial expressions based on the local features instead of processing first the global form, a more common strategy in TD individuals [74, 4, 5]. In other words, when looking at facial stimuli autistic individuals tend to give more weight to the bottom-up configural processing over top-down information [75]. According to our results, a possible mechanism for the beneficial effects of OT is its ability to shift the balance from bottom-up processing toward a more top-down-oriented perception. This shift, which occurs, to some extent, in both social and non-social stimuli might be more relevant in facial processing where gestalt integration is preferred. During the presence of a face that requires emotional interpretation, it is possible that the reduction of gamma connectivity, following OT administration, decreased the cognitive weight of the bottom-up information transference from the local features and, together with the overall increase in top-down related connectivity, allowed for better processing of the presented information.

The correlation between low-gamma connectivity and improved social perception in the OT trials can be explained also in terms of the excitation-inhibition (*E*/*I*) model, where the signal-to-noise neural ratio is manifested in the activity of gamma and its relationship to alpha power [44, 76]. Shifts in this ratio toward a noisy neural transference may underpin ASD deficits during social and emotional processing [29, 77, 78]. Our results suggest a new perspective for interoperate OT effects, suggesting that during visual perception, exogenous OT strengthens the neural ability for posterior-frontal adaptive information transference by reducing the signal-to-noise ratio. This interpretation corresponds to Lopatina et al. (2018) theoretical paper suggesting that OT influences *E*/*I* balance by modulating gamma-band activity. We show here that OT not only modulates gamma band but also influences alpha and beta frequencies which had a crucial role in visual attention. At the same time, although it has been shown that long-distance synchronization in low gamma (around 40 Hz) occurs during face processing [79, 55], gamma connectivity as a “neural binding” mechanism is mostly discussed at the local/synaptic level [80]. Thus, although our results present an important modulation of low and high coherence simultaneously, to establish the influence of OT on the *E*/*I* ratio in ASD at a more global level, future studies should examine the relationship between gamma and lower frequencies using cross-frequency methods such as phase–amplitude coupling [41]. We treat the present study as a preliminary study and encourage further studies to explore the specific effects of OT on high-frequency bands during social interpretation more thoroughly. Moreover, it should be remembered that this proposed model is theoretical, and further studies which allow direct measurement of excitatory and inhibitory intracellular processes are required to confirm our hypothesis regarding the relationship between OT effects and the *E*/*I* model.

In recent years, there is a debate in the literature regarding the prolonged influences of OT in individuals with ASD. While former studies show that some individuals with ASD can benefit from the administration of OT, in their comprehensive meta-analysis Sikich and colleagues [81] suggest that over time the effects of OT on common social behaviors are not significant. The current study on the other hand highlights the acute influence of OT in adolescents with ASD, Namely, in line with Geschwind’s response [82] to the mentioned paper, we suggest that acute administration of OT enables a neural basis that, in combination with treatments that provide an opportunity to learn, has the potential to promote the processing of social information during treatment in individuals with ASD. Following Ford and Young’s [83] opinion, which highlights the subtle and context-dependent influences of OT, we suggest that acute administration of OT can open a window of time in which the early perceptual stages will be more global-oriented and top-down information will be more meaningful or available to the individual. Specifically, in our opinion, OT can be a good pairing with cognitive treatments that work on the identification of social cues or social-oriented therapy that focuses more on interpretations and appropriate responses during social situations. Using OT as an augmentation tool in this manner can allow for more effective learning of social stimuli during interventions and reduce the time required to achieve the therapeutic goals. Furthermore, our results suggest that OT increases relevant activity more in those with higher capabilities, as can be seen in the positive correlation between OT effects and the ADOS score. This could offer a path to possible new interventions, that has not always existed, for autistic people with higher social capabilities. 

### Limitations

Although our study provides new insights regarding the influence of OT on the early modulation of neural processes between social-related regions in ASD, it has several important limitations. First, our sample was composed of only 24 adolescents. A number of parameters limited the choice of the current sample selection. Namely, we focused on youth (ages 12–18) males only with high functional abilities. Moreover, all individuals were required to come to the laboratory twice (or three times if the clinical diagnosis needed to be renewed). In addition, our paradigm, which consists of the administration of OT, imaging sessions, and performance tasks, has led to long and complex meetings. Although the size of our sample is equivalent to the sample sizes from other studies with similar experimental designs [27, 29], given the complexity of the electrophysiology analyses and the neural variability that exists between individuals, we encourage follow-up studies to examine the effects of OT on early attentional processes on a more extensive and heterogenic sample in terms of gender, age, and clinical characteristics. Our sample size can also explain the lack of correlation between OT effects and the age or clinical assessment of the participants. Thus, another advantage of a larger and more diverse sample is identifying the individual who could benefit the most from OT administration.

## Conclusions

The current study present the influences of OT on early neural dynamics and connectivity patterns between the fusiform and medial frontal regions during social and non-social processing in ASD. By showing that OT impacts bottom-up and top-down modulation, in a different direction, immediately after the appearance of stimuli, we suggest a new and refined neural mechanism that can explain the social-related effects of OT in individuals with ASD during the earliest stages of processing. Alongside the possible therapeutic implication for OT in ASD during visual perception, our results also contribute to the growing understanding of the relationship between atypical connectivity in adolescents with ASD and their ability to perceive social and non-social stimuli in the world.

## Supplementary Information

**Additional file 1.** Medication information.

## Data Availability

Due to the clinical features and the age of our sample as well as the sizes and nature of the imaging files, all data will be sent upon request. Please send an e-mail to the corresponding author for more details.

## Abbreviations

ADOS: Autism Diagnostic Observation Schedule
ANOVA: Analysis of variance
ASD: Autism spectrum disorder
*E*/*I*: Excitation/inhibition
EEG: Electroencephalography
ICA: Independent component analysis
LCMV: Linearly constrained minimum variance
MEG: Magnetoencephalography
MIE: Mind in the eyes
mPFC: Medial prefrontal cortex
MRI: Magnetic resonance imaging
OT: Oxytocin
PL: Placebo
RMET: Reading the mind in the eyes task
ROI/s: Region/s of interest
TD: Typically developed
WASI: Wechsler Abbreviated Scale of Intelligence
