# Supplementary material for: Oxytocin impacts top-down and bottom-up social perception in adolescents with ASD: a MEG study of neural connectivity
Source: Mol Autism. 2022 Sep 5;13:36. doi: 10.1186/s13229-022-00513-6 (PMC9446859; doi:10.1186/s13229-022-00513-6)
Supplement: Supplementary file 1 — Additional file 1. Medication information. [file 13229_2022_513_MOESM1_ESM.docx]

**Supplementary information**

**Medication information**

Due to the high comorbidity between ASD and other neurological symptoms, medical treatment was not an excluded criterion. However, each individual's treatment was examined independently to evaluate the possible influence on the outcomes. A doctor accompanied the experiment, and each medicated participant was approved depending on the type of drug and the medical doses the individuals received.

The following are the names of the drugs whose use did not constitute a restrictive condition for participation in the trial.

- Fluoxetine (Flutine, Prizma)
- Strattera
- Risperdal (Risperidone)
- Adderall
- Aripiprazole
- Lisdexamfetamine (Vyvanse)
- Methylphenidate (Ritalin)
- Montelukast (Singulair)
- Gerdon
- Genotropin
- Escitalopram (Cipralex)
